# The Sulfilimine Analogue of Allicin, *S*-Allyl-*S*-(*S*-allyl)-*N*-Cyanosulfilimine, Is Antimicrobial and Reacts with Glutathione

**DOI:** 10.3390/antiox9111086

**Published:** 2020-11-04

**Authors:** Tobias Horn, Wolfgang Bettray, Ulrike Noll, Felix Krauskopf, Meng-Ruo Huang, Carsten Bolm, Alan J. Slusarenko, Martin C. H. Gruhlke

**Affiliations:** 1 Department of Plant Physiology, RWTH Aachen University, 52056 Aachen, Germany; tobias.horn@RWTH-aachen.de (T.H.); noll@bio3.rwth-aachen.de (U.N.); alan.slusarenko@bio3.rwth-aachen.de (A.J.S.); 2 Institute of Organic Chemistry, RWTH Aachen University, 52056 Aachen, Germany; Bettray@RWTH-Aachen.de (W.B.); felix.krauskopf@rwth-aachen.de (F.K.); d03223203@ntu.edu.tw (M.-R.H.); Carsten.Bolm@rwth-aachen.de (C.B.)

**Keywords:** allicin analogue, *N*-cyanosulfilimine, glutathione reductase

## Abstract

When cells of garlic (*Allium sativum*) are disrupted by wounding, they produce the defense substance allicin (diallylthiosulfinate). Allicin is an efficient thiol trap and readily passes through cell membranes into the cytosol, where it behaves as a redox toxin by oxidizing the cellular glutathione (GSH) pool and producing *S*-allylmercaptoglutathione (GSSA). An *N*-cyanosulfilimine analogue of allicin (CSA), which was predicted to have similar reactivity towards thiol groups but be more stable in storage, was synthesized and its properties investigated. Similarly to allicin, CSA was shown to inhibit the growth of various bacteria, a fungus (baker’s yeast), and *Arabidopsis* roots. A chemogenetic screen showed that yeast mutants with compromised GSH levels and metabolism were hypersensitive to CSA. GSH reacted with CSA to produce allyltrisulfanylglutathione (GS_3_A), which was a white solid virtually insoluble in water. Yeast *Δgsh1* mutants are unable to synthesize GSH because they lack the γ-glutamylcysteine synthetase (*GSH1*) gene, and they are unable to grow without GSH supplementation in the medium. GS_3_A in the growth medium supported the auxotrophic requirement for GSH in *Δgsh1* mutants. This result suggests that GS_3_A is being reduced to GSH in vivo, possibly by the enzyme glutathione reductase (GR), which has been shown to accept GSSA as a substrate. The results suggest that CSA has a mode of action similar to allicin and is effective at similar concentrations.

## 1. Introduction

**Scheme 1:**
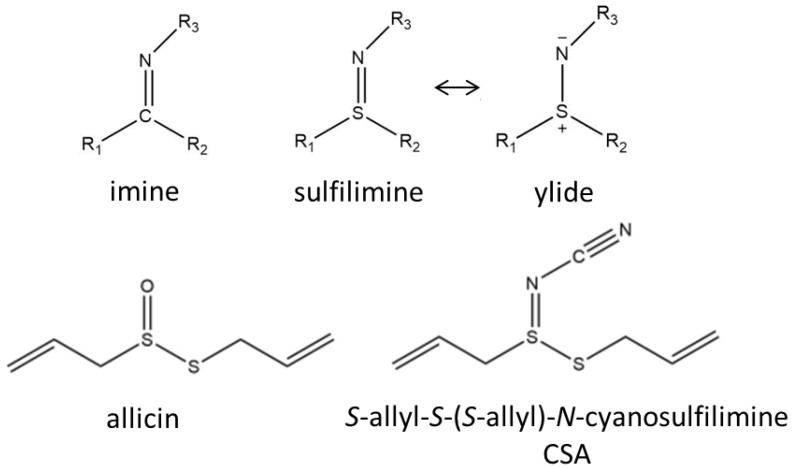
Shows the imine/sulfilimine structures and the mesomeric sulfilimine/ylide forms, as well as the structural formula of allicin and the *N*-cyanosulfilimine allicin analogue CSA (*S*-allyl-*S*-(*S*-allyl)-*N*-cyanosulfilimine).

Sulfilimines are a group of organic sulfur compounds in which the sulfur atom is linked to a nitrogen atom via a formal double bond (Scheme 1). In the literature such structures have also been named sulfimides, sulfilimides, or iminosulfurans [1]. The sulfilimine molecule is mesomerically stabilized and in the ionic form can be regarded as a sulfur/nitrogen ylide (Scheme 1) [2]. There is much interest in sulfilimines because they have been discussed as potential anticancer drugs [3], and ylides are the starting point for many organic syntheses. The stability of sulfilimines is greatly influenced by the nature of the R_3_ substituent group; thus, electronegative carbonyl, sulfonyl, or cyano groups stabilize the S=N bond. In this context, *N*-cyanosulfilimines proved particularly interesting, and, consequently, their synthetic accessibility has recently been greatly improved [4,5,6]. Furthermore, significant bioactivities of such compounds have been revealed [7,8].

Allicin (*S*-prop-2-en-1-yl prop-2-ene-1-sulfinothioate, Scheme 1) was shown by Cavallito to be the antimicrobial substance produced by garlic [9,10], and it was later demonstrated to deplete the cellular GSH pool, react with bacillothiol in Gram positive Bacillus, and *S*-thioallylate accessible cysteines in proteins [11,12,13]. Allicin is produced by damaged cells in which the enzyme alliinase comes together with its substrate alliin, both of which are usually separately compartmentalized in the cell. Allicin is a major secondary metabolite produced by garlic and a single clove can yield up to 5 mg [14]. Allicin gives fresh garlic its characteristic odor [15]. Allicin has been shown to kill many pathogenic bacteria in vitro, even as a vapor [16,17,18]. The electron-withdrawing nature of the *O*-atom in the thiosulfinate group in allicin leads to an electrophilic sulfur center, which reacts readily with thiols without the need for enzymic catalysis [11,19,20]. Furthermore, allicin can react with accessible cysteine thiols in proteins by *S*-thioallylation causing disulfide stress in cells [13,21,22].

Previously, we investigated the properties of thiosulfinate analogues of allicin by varying the alkyl groups attached to the thiosulfinate functional group. Here, we report the replacement of the thiosulfinate functional group in the synthesis of the *S*-allyl-*S*-(*S*-allyl)-*N*-cyanosulfilimine analogue of allicin (CSA), which, to our knowledge, has never been synthesized before. Because of the electron-withdrawing properties of the cyanosulfilimine group, we predicted that CSA would also have an electrophilic sulfur-center and be a potent thiol-trapping reagent similar to allicin but with a better thermal stability. Therefore, the antimicrobial activity of CSA was investigated and a yeast-based chemogenetic screen was performed to gain an insight into its potential mode of action [23].

Glutathione (GSH) is the major low molecular weight thiol in most eukaryotic and many prokaryotic cells, and it buffers and protects cells against oxidative stress [24,25] and is an electron donor for several reducing enzyme systems. GSH metabolism and the GSH pool have been shown previously to be particularly important in protecting cells against allicin action [26,27,28] and, thus, might be expected to similarly protect cells against the effects of CSA.

**Scheme 2:**
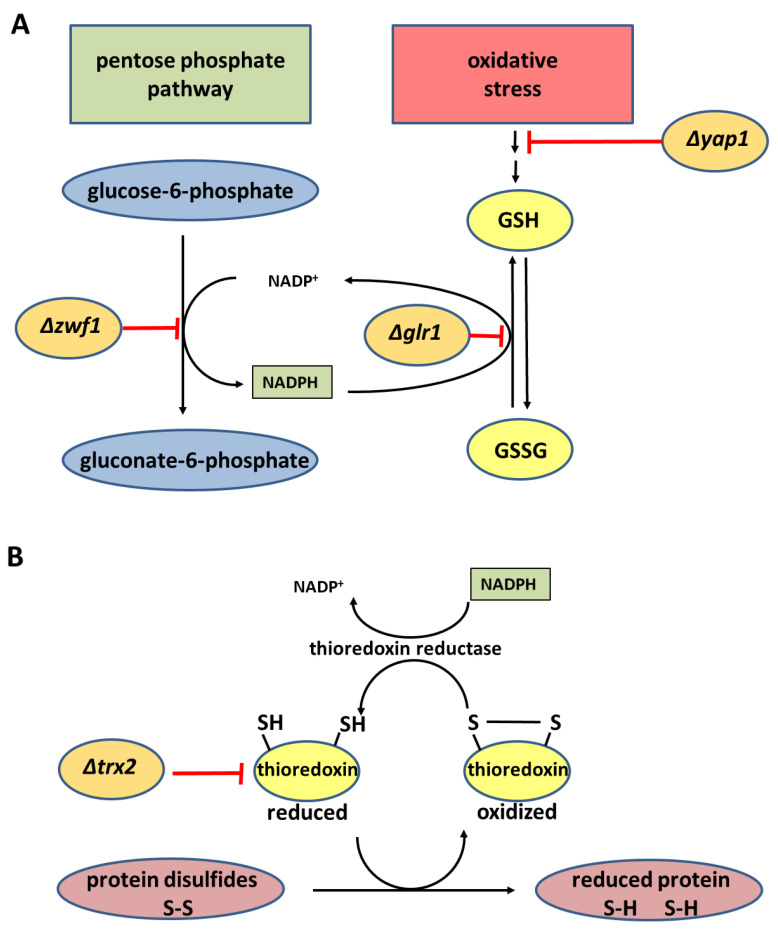
(**A**) The positions where the *Δyap1*, *Δzwf1*, and *Δglr1* mutants affect glutathione synthesis and metabolism. (**B**) The effect due to lack of thioredoxin in the *Δtrx2* mutant in compromising the reduction by thioredoxin-dependent mechanisms of protein disulfides produced under oxidative stress conditions. NADPH produced in the pentose phosphate pathway serves as a reductant for both glutathione reductase and thioredoxin reductase.

Under oxidative stress conditions GSH dimerizes to glutathione disulfide (GSSG). Furthermore, cysteine thiols in proteins may be reversibly glutathiolated to protect them from over-oxidation [21]. The GSH pool within the cell is restored by the reduction of GSSG to GSH by the NADPH-dependent enzyme glutathione reductase (Glr, E.C. 1.8.17) [24]. Glr1 not only reduces GSSG to GSH but also can accept the reaction product of allicin and glutathione (*S*-allylmercaptoglutathione, GSSA) as a substrate [11,12]. Scheme 2 shows the positions where the various mutants tested affect glutathione synthesis and metabolism. The Yap1 transcription factor coordinates the oxidative stress response (OSR) in yeast, including the activation of the *Gsh1* gene for the rate-limiting Gsh1 enzyme in GSH synthesis. The Yap1p transcription factor activates several genes, enabling challenged cells to resist the effects of oxidative stress. Thus, as well as genes on the GSH biosynthetic pathway, glutathione reductase, glutaredoxins, thioredoxins, and thioredoxin reductase genes are activated. As a result of oxidative stress, protein disulfides form from cysteine thiols in proteins. The small (~12 kDa) protein thioredoxin reduces protein disulfides and, in doing so, forms an internal disulfide bond between two cysteines. Thioredoxin reductase regenerates thioredoxin by using NADPH as a reductant to reduce the disulfide bond. Thus, the production of NADPH is of major importance in providing reducing equivalents to protect against oxidative stress and the pentose phosphate pathway (PPP) is the major source of NADPH in eukaryotic and prokaryotic cells. Zwf1 (glucose-6-phosphare dehydrogenase) catalyzes the first step in the PPP (Scheme 2). In addition to serving as a reductant for protein disulfides, thioredoxin2 is important for resistance to H_2_O_2_-derived oxidative stress [29,30,31,32].

The goal of this investigation was to test whether CSA, which although based on the allicin molecule no longer retains the thiosulfinate functional group, has antimicrobial properties and whether its mode of action might be similar to that of allicin.

## 2. Materials and Methods

### 2.1. Synthesis and LC-MS of S-allyl-S-(S-allyl)-N-cyanosulfilimine

The synthesis followed a reaction protocol developed for sulfide imidations [4].

Into a 15 mL sealed-tube were added 0.39 mL of diallyl disulfide (with a technical grade of 80%; Sigma-Aldrich, Steinheim, Germany; 365.7 mg, 2.5 mmol, 1.0 equiv) and 2.5 mL of MeCN. Under stirring at room temperature, cyanamide (375 mg, 9.0 mmol, 3.6 equiv) and (diacetoxyiodo)benzene (2.67 g, 8.4 mmol, 3.3 equiv) were added to the reaction mixture in three portions every 40 min. The reaction was monitored by thin-layer chromatography (TLC). After consumption of the starting material, the solvent was removed by evaporation, and the product was purified by flash column chromatography over silica (pentane to pentane:dichloromethane 4:1), yielding 223 mg (1.20 mmol, 60% based on the listed purity of the starting material of 80%) of the product (CSA) as a colorless liquid. ^1^H-NMR (400 MHz, CDCl_3_) δ = 5.95–5.80 (m, 2H), 5.42–5.24 (m, 4H), 3.89 (d, J = 7.0 Hz, 2H), 3.71 (d, J = 7.0 Hz, 2H) ppm; ^13^C{^1^H}-NMR (101 MHz, CDCl_3_) δ = 132.1, 131.0, 121.9, 120.6, 116.1, 59.6, 42.8 ppm; IR (ATR) ν [cm^–1^] = 3367 (vw), 3084 (w), 2981 (w), 2923 (w), 2660 (vw), 2330 (vw), 2210 (vs), 2090 (vw), 1994 (vw), 1857 (vw), 1738 (vw), 1636 (m), 1571 (vw), 1422 (s), 1338 (m), 1288 (m), 1222 (m), 1170 (m), 1127 (m), 1078 (w), 987 (vs), 927 (vs), 721 (s); MS (EI, 70 eV): *m/z* (%) = 187.0 (3), 144.9 (12), 121.0 (100), 73.0 (12), 45.2 (11); high-resolution mass spectrometry HRMS (ESI): C_7_H_10_N_2_NaS_2_ Calcd. 209.0178 Found 209.0173.

### 2.2. Stability of CSA Compared to Allicin

Aqueous 20 mM CSA and allicin solutions: (1.5 mL) each were transferred to a reaction vessel and incubated at 100 °C in a heating block. A sample was taken every 10 min, and the relative amount of substance was quantified by high-pressure liquid chromatography (HPLC). Separation was performed using H_2_O as mobile phase A and methanol as mobile phase B with the following gradient: 56% A (pre-run); 53% A (10 min); 7% A (15 min); 7% A (30 min); 56% A (31 min); 56% A (35 min) at a flow rate of 1 mL/min on a C18-reverse phase column. The detection was performed with a UV detector (Jasco GmbH, Groß Umstadt, Germany) at 254 nm.

### 2.3. Reaction of GSH with CSA and LC/MS of the GS_3_A Product

l-glutathione (400 mg, Carl Roth, GmbH, Karlsruhe, Germany) was dissolved in up to 4 mL of distilled H_2_O. CSA (280 mg) were dissolved in up to 12 mL in methanol. Under stirring, the methanolic solution was slowly added to the aqueous solution, and, subsequently, the stirring was continued for 2 h at room temperature. The insoluble product was collected by filtration and washed with 3 × 5 mL of dichloromethane and finally with 5 mL of distilled H_2_O. Subsequently, the product was dried to constant weight at 37 °C.

ESI-MS measurements were performed on a Thermo Fisher Scientific Orbitrap XL mass spectrometer (Bremen, Germany) in high resolution FT-mode. The sample was diluted in acetonitrile (AcCN)/Water (H_2_O) *v/v* 50:50 with HCl-solution added and introduced via direct flow using a syringe pump with a flow of 5 μL/min. 

### 2.4. Experiments with Microorganisms

*Bacillus subtilis*, *Escherichia coli*, and *Photorhabdus luminescens* were cultivated on LB medium 5 g/L yeast extract (Duchefa, Haarlem, Netherlands), 10 g/L peptone (Duchefa, Haarlem, Netherlands), 10 g sodium chloride (Applichem, Darmstadt, Germany), and 1.5% agar (Carl Roth, Karlsruhe, Germany). The pseudomonads (*Pseudomonas fluorescens* AR-1, an allicin resistant strain and *Pseudomonas syringae* 1446A) were cultivated on King’s B medium (20 g/L peptone (Duchefa, Haarlem, Netherlands), 1.5 g/L anhydrous K_2_HPO_4_ (Applichem, Darmstadt, Germany), 12.6 g/L glycerol (Applichem, Darmstadt, Germany), and 1.5% agar (Carl Roth, Karlsruhe, Germany). With the exception of *E. coli*, which were cultivated at 37 °C, all bacteria were cultivated at 28 °C. 

The plate inhibition assay was performed by diluting cultures overnight to an OD_600_ of 0.2. One hundred and twenty microliters of these cultures were plated onto a 20 mL LB agar plate (Pseudomonas: 20 mL KB agar plate) and uniformly distributed on the plate using sterile glass beads (ø = 4 mm). The test was evaluated after 48 h of cultivation under appropriate conditions as described above. 

The haploid *Saccharomyces cerevisiae* yeast strain BY4742 (Matα; his3∆1; leu2∆0, lys2∆0, ura3∆0) [33] was used. The BY4742 mutant ∆*gsh1* (Y17097) used in this study lacks the gene for γ-glutamylcysteine synthetase (YJL101C), which catalyzes the first step in glutathione biosynthesis. The mutant was obtained from the EUROSCARF Collection, University of Frankfurt (Main), Germany (http://www.euroscarf.de/).

Yeast was grown in complete synthetic mixture (CSM) medium (0.79 g L^−1^ CSM Drop-Out: Complete (ForMedium, Norwich, UK); 6.9 g L^−1^ Yeast Nitrogen Base (ForMedium, Norwich, UK); 40 g L^−1^ d-Glucose (Carl Roth, Karlsruhe, Germany); and 15 g L^−1^ agar for solid medium.

### 2.5. Experiments with Arabidopsis Roots

The *Arabidopsis* seedling root assay was performed after Reference [34]. Surface-sterilized *Arabidopsis thaliana* seeds (Col-0, *pad2* and *gr1*) were sown on Murashige & Skoog (MS) solid medium and were grown under short day conditions (8 h light) at 21 °C. The Petri plates were tilted to an angle of approximately 70° to ensure root growth according to root gravitropism. After three days of cultivation, seedlings were transferred to MS medium that contained different amounts of CSA or allicin. After three days treatment, seedlings were photographed, and the root length was measured.

## 3. Results and Discussion

### 3.1. Stability of CSA Compared to Allicin

**Figure 1:**
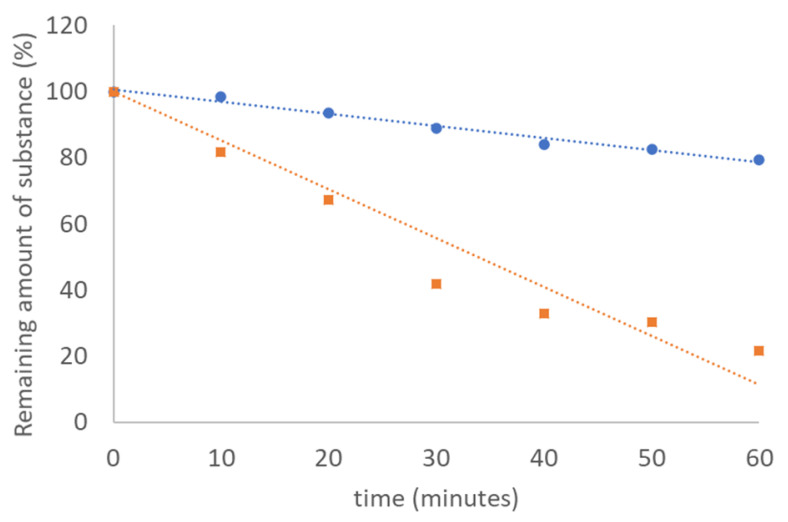
Comparative stability of allicin and CSA at 100 °C over a period of one hour. The relative amount of substance was determined by HPLC.

CSA is an analogue of allicin, which, however, does not have a thiosulfinate as a functional group, like allicin, but a sulfilimine group (Scheme 1). Allicin is a highly potent antibiotically active substance, but has relatively low thermal stability. To test whether CSA offers an advantage over allicin in this respect, both substances were incubated at 100 °C for one hour, and the remaining amount of substance was determined using HPLC. It is shown that allicin is degraded very quickly, as is already known, whereas CSA is more persistent in terms of thermal stability (Figure 1). This means that there is an advantage of CSA over allicin in this respect, and we, therefore, investigated its antibiotic effectiveness. 

### 3.2. CSA Inhibits the Growth of Bacteria and Yeast in Agar-Diffusion Tests

In agar diffusion assays, solutions of a test substance are applied to wells and the substance diffuses into the agar medium giving a gradient of decreasing concentration away from the well. The test organisms are applied evenly over the Petri plate and can grow up to the threshold inhibitory concentration around each well. Thus, the relative sensitivities of various microorganisms to a given test substance can be demonstrated. However, the relative effectivities of different test substances against a specific microorganism cannot be compared in this assay because of the different diffusion behavior of the test molecules. To compare the relative effectivities of different test substances, fixed concentrations are incorporated into the growth medium and a dilution series of a cell-suspension culture pipetted onto the medium in a so-called ‘drop test’ [23], as in Section 3.3.

**Figure 2:**
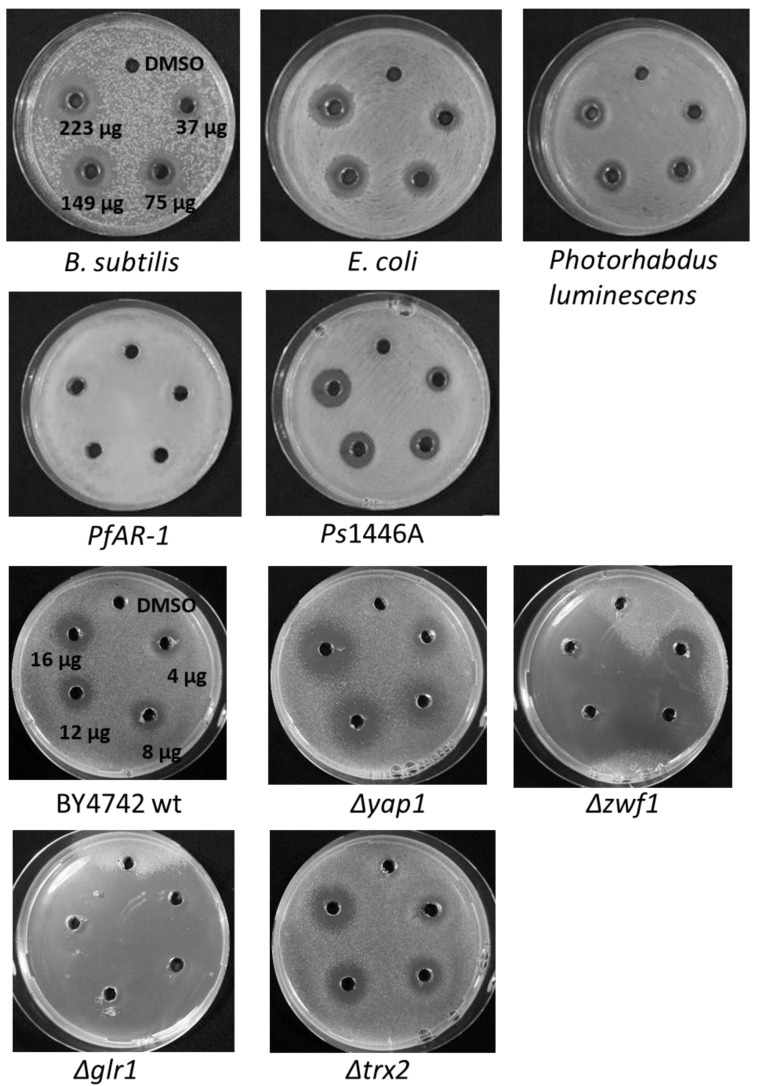
CSA (*S*-allyl-*S*-(*S*-allyl)-*N*-cyanosulfilimine) inhibits the growth of bacteria and yeast. Bacteria were spread on the surface of the agar medium and 20 µL of 10, 20, 40, and 60 mM CSA in dimethylsulfoxide (DMSO) pipetted into wells cut out of the agar, giving total amounts of 37, 75, 149, and 223 µg CSA, respectively. For the wildtype yeast strain BY4742 and the *Δyap1*, *Δzwf1*, *Δglr1*, and *Δtrx2* mutants, 20 µL of 1, 2, 3, and 4 mM CSA in DMSO was used, giving total amounts of 4, 8, 12, and 16 µg, respectively. The control was 20 µL of DMSO.

*Bacillus subtilis* (Gram positive), *Escherichia coli*, *Photorhabdus luminescens*, and *Pseudomonas syringae* pv. *phaseolicola* 1446A (all Gram negative) showed a dose-dependent inhibition of growth in the presence of CSA. In contrast, the highly allicin-resistant *Pseudomonas fluorescens* isolate *Pf*AR-1 showed only an extremely small inhibition zone at the highest concentration of CSA tested (Figure 2). *Pf*AR-1 was isolated from garlic and has 3 genomic islands carrying repeats containing genes which confer allicin resistance [28]. The insensitivity of *Pf*AR-1 to CSA, as well as allicin, suggests that the mode of action of both compounds might be similar, i.e., targeting the GSH pool and causing oxidative disulfide stress [13,27,28]. Instead of GSH, *Bacillus* spp. contain bacillothiol as a redox-buffer and protectant against oxidative stress. We have previously shown that allicin targets bacillothiol, and, presumably, this is similarly the case for CSA [13].

As previously demonstrated for allicin, the fungus *Saccharomyces cerevisiae* (baker’s yeast) was much more sensitive to CSA than were bacteria, and comparable inhibition zones were observed with much lower amounts of active substance (Figure 2). 

The behavior of the *Δyap1*, *Δzwf1*, *Δglr1*, and *Δtrx2* yeast mutants to CSA was also similar to that for allicin [27], suggesting that CSA behaves similarly in cells by targeting thiols. Thus, as for allicin, the *Δyap1*, *Δzwf1*, and *Δglr1* mutants were all hypersensitive to CSA compared to wildtype cells, while the effect of the *Δtrx2* mutation was marginal. The lack of hypersensitivity in the *trx2* mutant suggests that, as for allicin, H_2_O_2_-generation is not an important factor in the oxidative stress caused by CSA. The Yap1 transcription factor coordinates the oxidative stress response in yeast, and *Δyap1* yeast cells were more sensitive to CSA than the wildtype, indicating that as for allicin, CSA caused oxidative stress in the cells. It was previously shown that allicin directly targets specific cysteines in Yap1 [26], and it is likely that CSA has a similar mechanism of action. As previously reported for allicin, the *Δzwf1* and *Δglr1* mutants showed extreme sensitivity to CSA. Indeed, the growth of *Δglr1* was inhibited over almost the entire Petri plate. Thus, GSH metabolism appears to be important for protecting the cells against CSA, and this suggests that, similarly to allicin, CSA works by causing disulfide stress via the oxidation of thiols [22,27,28].

### 3.3. Chemogenetic Screen of CSA Compared to Allicin in the Yeast Mutants Δglr1, Δtrx2, Δyap1, and Δzwf1

The inhibitory activity of CSA was compared directly to that of allicin to assess the relative effectiveness of the two substances using a sensitive drop test where fixed concentrations of the test substance are incorporated into the medium and a dilution series of cells plated out [19]. Furthermore, the role of GSH metabolism and the oxidative stress response (OSR) in protecting yeast against CSA described in Section 3.2 was here examined in more detail in a chemogenetic screen with selected yeast mutants [27,35].

**Figure 3:**
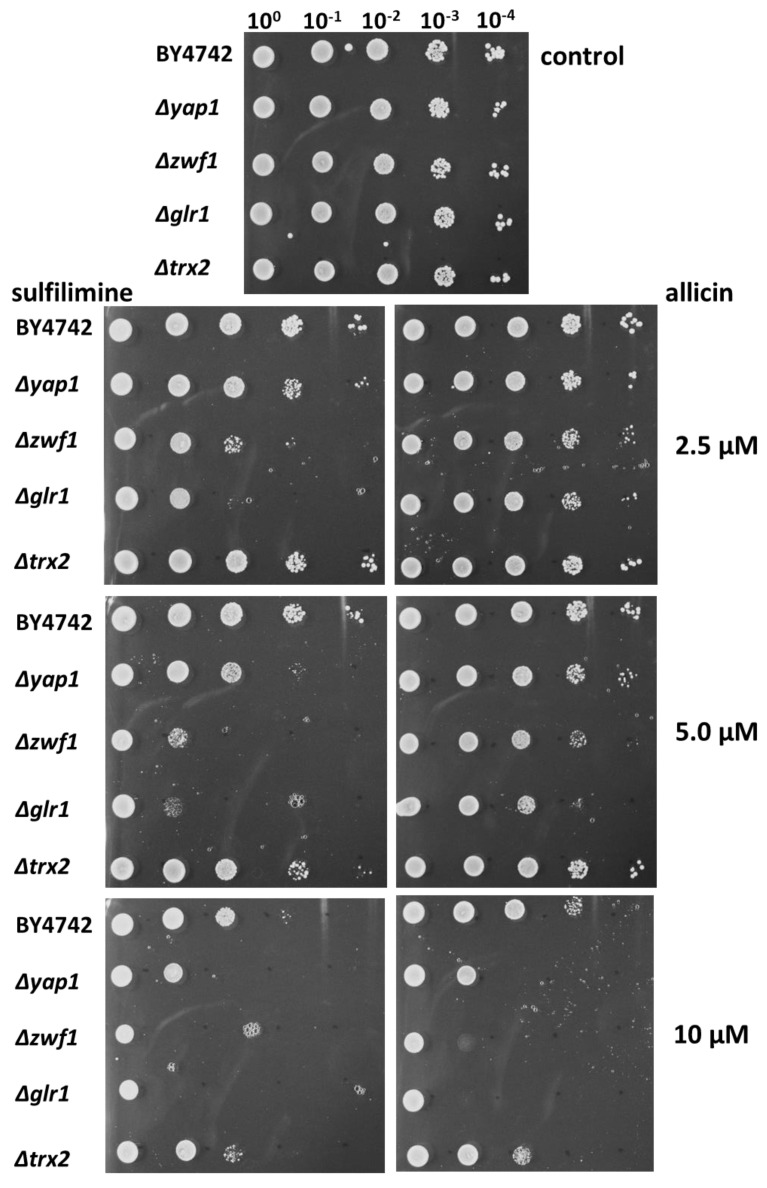
Chemogenetic screen of CSA (*S*-allyl-*S*-(*S*-allyl)-*N*-cyanosulfilimine) and allicin against a serial dilution of selected yeast mutants in a drop test.

In the absence of stress, the yeast BY4742 wildtype and the mutants all grew similarly down to the 10^−4^ dilution of the original culture (Figure 3). The BY4742 wildtype yeast strain showed similar sensitivity to both allicin and CSA over the 2.5–10 µM range; suggesting that the two compounds are similarly active on a mol-for-mol basis in yeast, with perhaps a slightly greater degree of inhibition visible at 10 µM CSA at the 10^−3^ dilution (Figure 3). However, although BY4742 was not inhibited by either CSA or allicin at 2.5 µM, the Δglr1 and Δzwf1 mutants were inhibited by CSA but not allicin at this concentration (Figure 3). The higher sensitivity of Δglr1, Δzwf1 and the Δyap1 mutant for CSA over allicin is very clear at 5 µM, but, at 10 µM, sensitivities are again similar (Figure 3). Taken together, these results suggest that CSA is slightly more active on an equimolar basis than allicin against yeast, but that the GSH and NADPH-based defence responses can cope equally well against oxidative stress caused by both compounds in the wildtype strain. 

The results emphasize the importance of GSH metabolism in overcoming CSA stress. The glutathione reductase (Glr) enzyme, that is absent in the Δglr1 mutant, uses NADPH as a cosubstrate and reductant. The major source of NADPH in the cell is the Zwf1 gene-encoded glucose-6-phosphate dehydrogenase, which catalyzes the first step in the pentose phosphate pathway (Scheme 2). Thus, the Δzwf1 mutant is compromised in the production of NADPH for Glr, and both the Δglr1 and Δzwf1 mutants showed a high degree of inhibition by CSA (Figure 3).

The OSR in yeast is coordinated by the Yap1p transcription factor, and it was shown previously that allicin activates Yap1 and that a Δyap1 mutant was hypersensitive to allicin [26]. The hypersensitivity of the Δyap1 mutant to CSA (Figure 2) shows that the protective OSR in yeast is needed for cells to work against the effects of CSA and implies that CSA, like allicin, causes oxidative stress in yeast.

It was previously shown that Trx2 plays only a minor role in protecting yeast cells against the effects of allicin [26,27], and this is also the case for CSA (Figure 3).

### 3.4. Effect of CSA on Arabidopsis Root Growth

**Figure 4:**
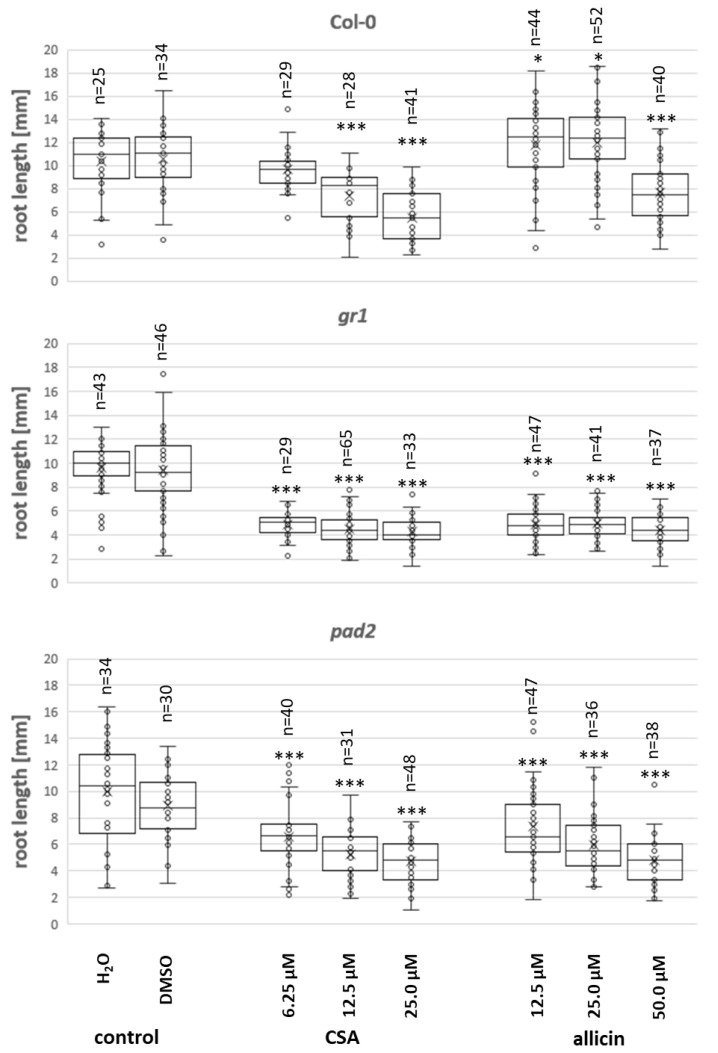
Effects of CSA and allicin on seedling root growth of *Arabidopsis thaliana* Col-0 (wildtype), a mutant with reduced glutathione (*pad2*), and a mutant lacking glutathione reductase (*gr1*). The box plots show the range of the data, the mean (x), the median (horizontal line), and standard deviation (box). A *t*-test of each sample-group against the untreated control was performed. For non-normally distributed data, the test was carried out on ranks (Wilcoxon–Mann–Whitney test): *: *p* < 0.05; ***: *p* < 0.001.

The importance of GSH levels and GSH metabolism in resistance against CSA was further supported by experiments using the model plant *Arabidopsis thaliana*. Arabidopsis Col-0 wildtype and *pad2* and *gr1* mutants in the Col-0 background were tested. The *pad2* mutant has only approximately 20% of the GSH level found in the wildtype [36]. The *gr1* mutant line is a knockout mutant of glutathione reductase and has a higher proportion of GSSG in the glutathione pool because it cannot reduce GSSG back to GSH [37]. Seeds were germinated for three days before transplanting onto medium containing CSA. Root length was measured after a further three days of growth. As can be seen in Figure 4, CSA impaired root growth in the Col-0 wildtype in a concentration-dependent manner. Wildtype Col-0 seedlings were clearly more sensitive to CSA than to allicin. Thus, significant root growth inhibition (*p* < 0.001) was observed at 12.5 µM CSA whereas significant inhibition occurred between 25–50 µM allicin and <25 µM allicin was slightly stimulatory (*p* < 0.05) to root growth in the wildtype. The *gr1* and *pad2* mutants were both very sensitive to CSA and allicin at concentrations of 6.25–12.5 µM (*p* < 0.001, Figure 4). These results further confirm the important role observed in yeast (Figure 2 and Figure 3), of GSH metabolism in countering CSA-induced oxidative stress.

### 3.5. Reaction of CSA with GSH and Characterization of GS3A as the Reaction Product

**Figure 5:**
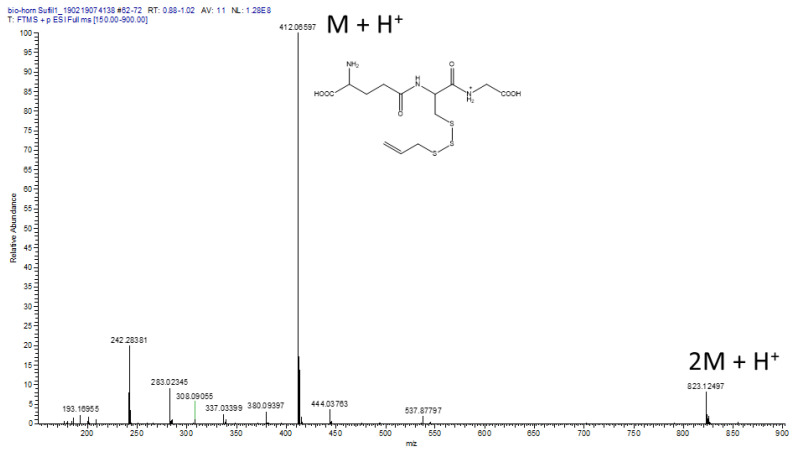
LC/MS of GS_3_A, the putative reaction product of CSA with GSH.

**Scheme 3:**
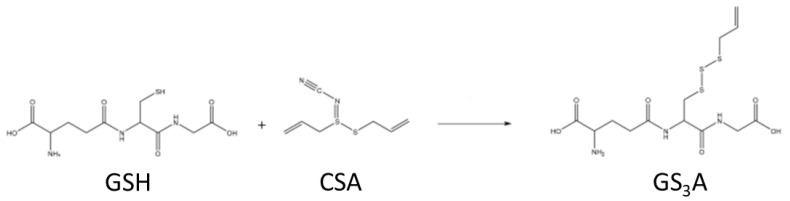
Shows the reaction of glutathione (GSH) with CSA to make allyltrisulfanylglutathione (GS_3_A).

CSA reacted with GSH at room temperature to produce a highly water-insoluble product. ESI-MS of this product (diluted in AcCN/H_2_O, HCl, measured on a Thermo Fisher Scientific Orbitrap XL) revealed a major signal at M + H^+^ = 412 Mass Units and a second signal for 2M + H^+^ = 823 Mass Units (Figure 5). HR-ESI-MS (Bremen, Germany) measurements proved the identity of the compound by exact mass measurements (HRMS (ESI): C_13_H_22_N_3_O_6_S_3_ Calcd. 412.06652 Found 412.06597). We have previously shown that allicin reacts with GSH to produce S-allylmercaptoglutathione (GSSA, M + H^+^ = 380). The reaction product of CSA with GSH has a mass compatible with an additional sulfur atom in the structure, i.e., allyltrisulfanylglutathione (GS_3_A, Scheme 3).

### 3.6. A Δgsh1 Mutant can Grow on Medium without GSH when Supplemented with GS_3_A 

**Figure 6:**
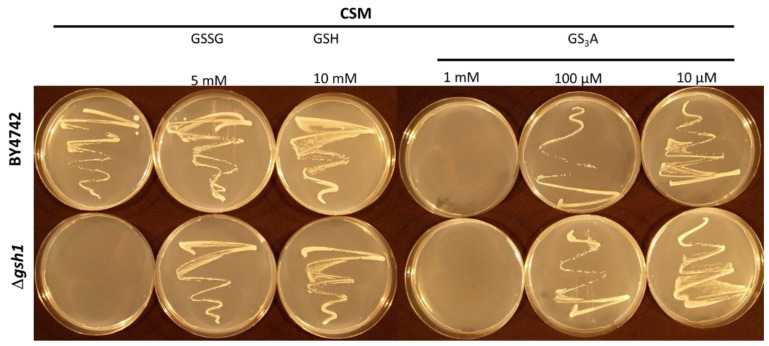
Growth of the wildtype diploid BY4742 yeast strain compared with the *Δgsh1* mutant on complete synthetic mixture (CSM) and CSM supplemented with 10 mM glutathione (GSH), 5 mM glutathione disulfide (GSSG), or 10 mM *S*-allylmercaptoglutathione (GSSA). A 100 µL aliquot of a logarithmically growing yeast culture adjusted to OD_600_ = 0.2 (OD = optical density) was spread onto the surface of each plate with an inoculation loop spatula.

*Δgsh1* yeast mutants lacking the Gsh1 enzyme (γ-glutamylcysteine synthetase) are auxotrophic for GSH, i.e., they cannot grow without GSH-supplementation. GSSG can serve as a source of GSH because it can be reduced by the NADPH-dependent enzyme glutathione reductase (Glr). In Figure 6 it can be seen that *S*-allyltrisulfanylglutathione (GS_3_A) can also serve as a source of GSH for the *Δgsh1* mutant, indicating that it is possibly a substrate for GR. Obviously, GS_3_A can inhibit growth of the diploid wildtype BY4742 yeast cells at 1 mM and appears to be partially inhibitory at 100 µM, while still being able to substitute for GSH at that concentration. At 10 µM GS_3_A, it does not appear to inhibit BY4742 cells, and it fully complements the *Δgsh1* mutant. Therefore, it seems likely that GS_3_A might also be a substrate for Glr. Trisulfides as substrates for Glr have been reported [38]. However, due to the extreme insolubility of GS_3_A we could not test this directly with GR in vitro. Nevertheless, this observation is of importance for in vivo considerations of the consequences of CSA reacting with GSH. Thus, at sublethal CSA doses, it seems that GS_3_A might be further metabolized by Glr, releasing GSH in the process.

## 4. Conclusions/Highlights

- CSA is antimicrobial in a dose-dependent manner.
- CSA probably has a similar mechanism of action to allicin.
- CSA reacts with GSH to make GS_3_A.
- GS_3_A can complement a *Δgsh1* yeast mutant and, therefore, may be a substrate for Glr.
